# Recent Advances in Biosynthesis and Bioactivity of Plant Caffeoylquinic Acids

**DOI:** 10.3390/cimb47110942

**Published:** 2025-11-13

**Authors:** Hanqin Chen, Bo Pan, Shilong Zhang, Xin Li, Yuyao Zhang, Kang Gao, Dongliang Chen, Lili Wang, Tianhua Jiang, Chang Luo, Conglin Huang

**Affiliations:** 1 Institute of Grassland, Flowers and Ecology, Beijing Academy of Agriculture and Forestry Sciences, Beijing 100097, China; 2 School of Landscape Architecture, Beijing University of Agriculture, Beijing 102206, China

**Keywords:** chlorogenic acid, caffeoylquinic acids, biosynthesis, bioactivity, pharmacologicals

## Abstract

Caffeoylquinic acids (CQAs), a class of phenolic acid metabolites widely distributed in plants, encompass 15 positional isomers from mono- to tetra-esters, with 5-O-caffeoylquinic acid (5-CQA) as the predominant form. The biosynthesis of 5-CQA from phenylalanine proceeds through five primary pathways, which are finely regulated by environmental, hormonal, and transcription factors from families such as MYB, WRKY, and bHLH. These regulators control 5-CQA synthesis by binding specifically to the promoter regions of key structural genes, including PAL, 4CL and HCT/HQT. Subsequently, 5-CQA serves as a central precursor for the biosynthesis of other CQAs. In terms of bioactivity, CQAs possess remarkable pharmacological activities, encompassing antioxidant, antimicrobial, anti-diabetic, anti-inflammatory and anti-tumor properties. For instance, anti-inflammatory effects are demonstrated by the ability of 5-CQA to reduce key pro-inflammatory cytokines (e.g., TNF-α and IL-1β) and downregulate the TLR4/NF-κB pathway. The synergistic action of 5-CQA with ultraviolet-A reduced succinate-coenzyme Q reductase activity by approximately 72%, highlighting its potential to disrupt bacterial metabolism and combat antibiotic resistance. Furthermore, 3,4,5-triCQA exhibits potent anti-influenza virus activity, potentially through a mechanism distinct from existing neuraminidase inhibitors. Beyond medicine, CQAs show promise in light industry. They serve as antibiotic alternatives in livestock feed to enhance gut health, extend food shelf life through their antioxidant activity, and function as active ingredients in UV-protective skincare formulations. CQAs also enhance plant stress tolerance to cold, arsenic, and pests by mechanisms such as scavenging reactive oxygen species and inhibiting pest mobility. While this review consolidates progress in the biosynthesis and bioactivity of CQAs specifically with caffeoyl substituents, future efforts should leverage modern biotechnological tools and interdisciplinary approaches to bridge critical knowledge gaps in their biosynthesis, transport, and clinical translation.

## 1. Introduction

Plant phenolics, as one of the most widespread and diverse classes of natural products, play indispensable roles in human health and plant defense. Among them, Caffeoylquinic acids (CQAs)—esters formed between quinic acid and caffeic acid—stand out for their significant pharmacological value and biological prevalence. This family encompasses mono-, di-, tri-, and tetra- esters, with 5-O-caffeoylquinic acid (5-CQA) being the most abundant and prominent member. It is designated as a key quality control marker in the Chinese Pharmacopoeia for numerous traditional medicinal herbs, including *Lonicera japonica* Thunb., *Prunus mume* Siebold & Zucc., *Chrysanthemum morifolium* Ramat., and *Eucommia ulmoides* Oliv., as well as for proprietary Chinese medicines such as Yinhuang Oral Liquid, Zhongjiefeng Extract, Yinzhihuang Granules, and Shuanghuanglian Oral Liquid [1]. Plants synthesize 5-CQA as the initial product via the phenylpropanoid pathway. This compound is subsequently converted into other CQAs, including 3-CQA, 4-CQA, and various poly-caffeoylquinic acids. These secondary metabolites exhibit diverse pharmacological properties, including broad-spectrum antimicrobial, anti-inflammatory, antitumor, neuroprotective, and antioxidant activities. They also play significant roles in plant growth, development, and responses to abiotic and biotic stresses [2,3,4,5,6,7,8].

This review is structured to provide a comprehensive overview of CQAs, encompassing literature published up to October 2025, with a dual focus on biosynthesis and bioactivities of CQAs. Research has established five distinct biosynthetic pathways for 5-CQA, originating from phenylalanine, the end product of the shikimate pathway [9]. Numerous studies have identified the structural genes involved and their upstream regulators. For instance, a recent study in *L. japonica* identified the R2R3-MYB transcription factor LmMYB111, which activates the synthesis of CQAs and luteoloside by binding to the promoters of multiple genes, including *PAL*, *4CL*, and *MYB4* [10]. In another study, Luo et al. compared CQAs content and antioxidant activity across various sweet potato (*Ipomoea batatas* (L.) Lam.) genotypes, identifying the GARP-type transcription factor *IbGLK1*, which promotes CQAs biosynthesis by directly binding to the promoters of downstream structural genes [11].

Recent studies show that CQAs influence a wide range of applications, such as pharmacology, animal husbandry, food preservation, skin brightening, and plant stress resistance [9,12]. Substantial evidence from animal models confirms CQAs anti-inflammatory, antimicrobial, and neuroprotective effects, with underlying mechanisms increasingly elucidated [2,3]. In animal husbandry, CQAs—as a natural plant-derived compound—has garnered significant research attention as an antibiotic alternative; For instance, dietary supplementation with 5-CQA in broiler chickens effectively ameliorates intestinal health under high-density (HD) stocking stress [13].

Owing to its antioxidant, UV-protective, and skin-conditioning properties, CQAs exemplifies the broader role of polyphenols in dermatological protection [14]. As a potent antioxidant, CQAs mitigates cold stress damage in plants by scavenging reactive oxygen species (ROS) such as H_2_O_2_ and O_2_^−^ [15].

The impetus for this new review stems from the conceptual and methodological breakthroughs witnessed since 2020. The widespread adoption of plant metabolomics and transcriptomics has catalyzed an explosion of research into plant secondary metabolites, unearthing a wealth of new data on CQAs. Key advances include the identification of novel biosynthetic enzymes and regulatory transcription factors in non-model plant species, a deeper understanding of their multifaceted roles in plant–environment interactions, and the discovery of novel pharmacological mechanisms. However, this rapidly expanding body of knowledge has yet to be synthesized into a comprehensive review that critically evaluates these recent discoveries. This review therefore aims to consolidate the latest reports on CQAs biosynthesis and bioactivity, pinpoint critical research gaps, and champion the application of modern biotechnological tools to fully unlock the potential of CQAs in medicine, agriculture, and light industry.

## 2. Types of CQAs

**Figure 1:**
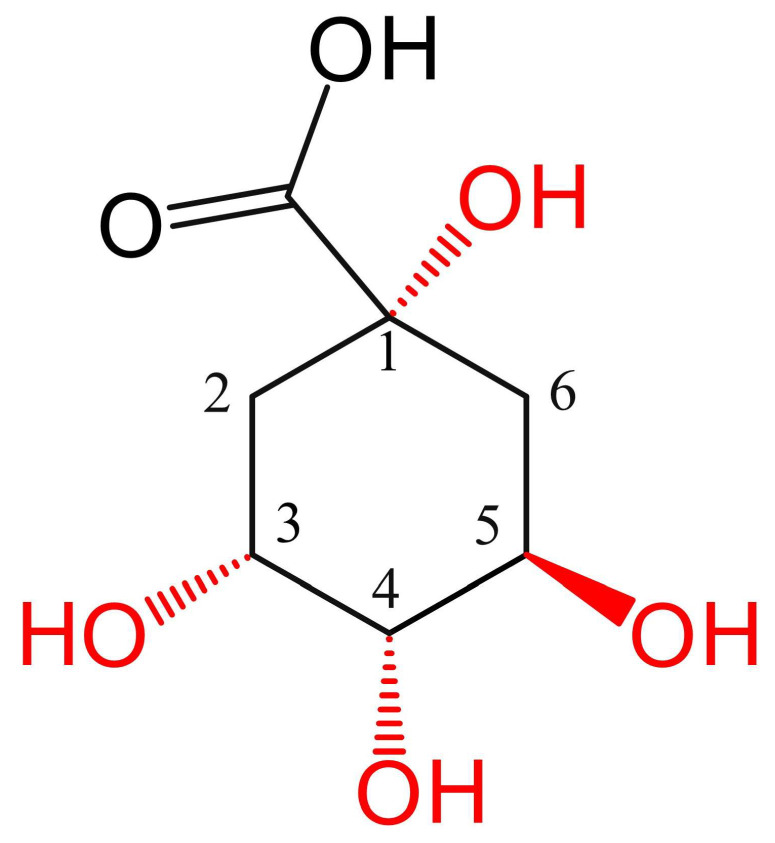
Chemical structures of quinic acid and four R-base derivatives. The stereochemistry of the natural (3R,5R)-configured quinic acid scaffold is crucial for activity and is retained in all derivatives, as highlighted by the wedged and dashed bonds. The numbers indicate the positional numbering of the substituents on the quinic acid ring, and the red color highlights potential substitution sites.

CQAs have multiple derivatives. Besides caffeic acid, the hydroxyl and carboxyl groups on the carbon ring of quinic acid ((1*S*,3*R*,4*S*,5*R*)-1,3,4,5-tetrahydroxycyclohexane-1-carboxylic acid) can form esters with other trans-cinnamic acids like ferulic acid, *p*-coumaric acid, and methyl esters [12]. The carbon ring of quinic acid contains four positions (1, 3, 4, 5) that undergo acylation with caffeoyl groups, as shown in Figure 1.

**Figure 2:**
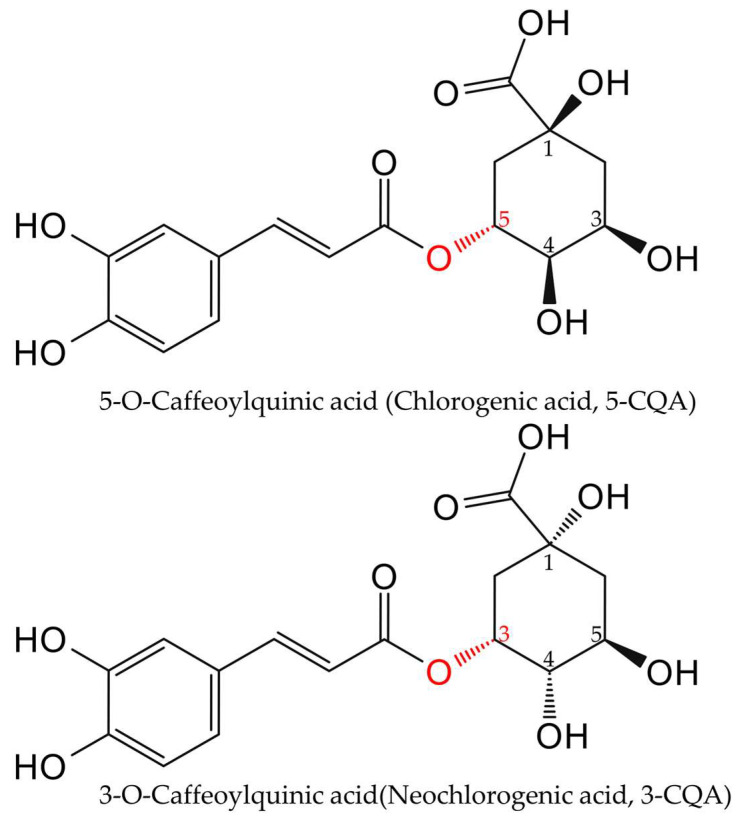
Key distinctions between 3-CQA (neochlorogenic acid) and 5-CQA (chlorogenic acid). This schematic highlights the critical stereochemical differences arising from their acylation sites, which are essential for resolving the persistent misapplication of the term “chlorogenic acid” in the literature.

A critical nomenclature clarification is essential, particularly regarding the distinct isomers 3-CQA and 5-CQA. To clarify these critical distinctions, we provide a direct comparison in Figure 2. Historically, “chlorogenic acid” referred to 3-CQA, but the 1976 IUPAC revision reassigned it to 5-CQA. This outdated usage persists in some literature and supplier catalogs, causing ongoing confusion. To ensure clarity throughout this review, we will exclusively use the systematic name 5-O-Caffeoylquinic acid (5-CQA), which corresponds to CAS number 327-97-9.

This review focuses exclusively on recent advances concerning caffeic acid substituents of the quinic acid backbone. The attachment of one to four caffeoyl groups to different hydroxyl positions of quinic acid generates a well-defined family of fifteen positional isomers. As summarized in Table 1, the CQA family comprises a total of 15 CQAs, ranging from mono- to tetra-caffeoyl substituted forms [16,17]. These CQAs exhibit distinct biological activities that generally correlate with the number of caffeoyl substitutions, following the order of tetra- > tri- > di- > mono-CQAs [9]. The table systematically lists the CAS registry numbers alongside the corresponding systematic names for each compound, thereby helping to mitigate potential confusion arising from historical or conflicting nomenclature in the literature.

## 3. Biosynthesis of CQAs

### 3.1. Five Biosynthetic Routes

**Figure 3:**
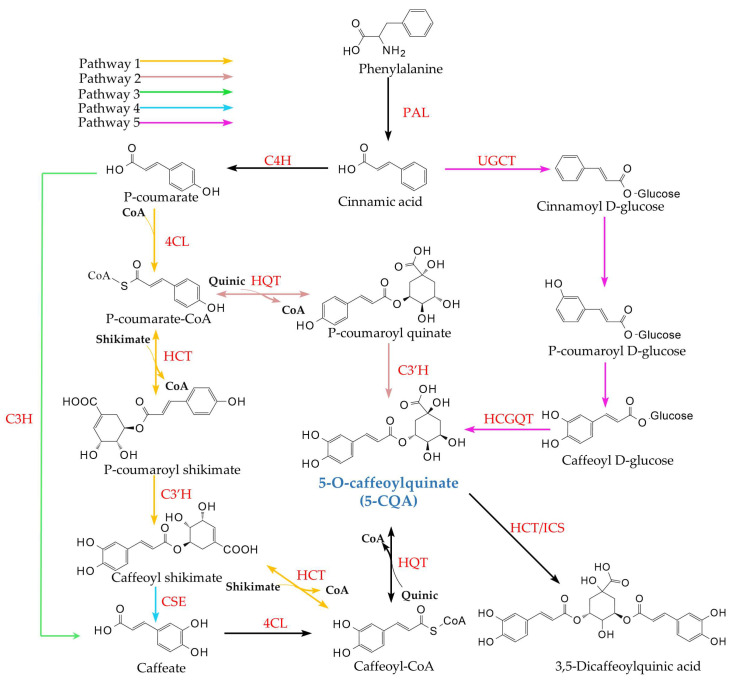
Different biosynthetic pathways leading to Plant CQAs. The five distinct 5-CQA biosynthetic pathways identified across different plant species are summarized here. Abbreviations: PAL, phenylalanine ammonia-lyase; C4H, cinnamic acid 4-hydroxylase; 4CL, 4-coumaric acid:CoA ligase; C3H, *p*-coumaric acid 3-hydroxylase; C3’H, *p*-coumaroyl-shikimic acid/quinic acid 3-hydroxylase; CSE, caffeoyl shikimic acid esterase; HCT, hydroxycinnamoyl-CoA:shikimic acid/quinic acid hydroxycinnamoyltransferase; HQT, hydroxycinnamoyl-CoA:quinic acid hydroxycinnamoyltransferase; UGCT, UDP-Glycosyltransferases; HCGQT, Hydroxycinnamoyl-D-g1ucose:quinate hvdroxvcinnam; ICS, isochlorogenic acid synthase.

Plants biosynthesize CQAs primarily via the phenylpropanoid pathway, which first yields 5-CQA [9]. This central compound then serves as a precursor for its isomers, such as 3-CQA and 4-CQA, as well as for more complex poly-caffeoylquinic acids. Furthermore, 5-CQA occupies a branch point in the pathway leading to lignin monomer synthesis, a connection that has been extensively documented [18]. Given this central role, the following section reviews five reported biosynthetic routes for 5-CQA. Gene expression within these pathways varies significantly across plant species. Notably, in many species, only one or two of these routes function as the predominant biosynthetic pathways for 5-CQA, while some are unique to particular plant lineages [19,20,21]. 5-CQA synthesis initiates when phenylalanine undergoes deamination by Phenylalanine ammonia-lyase (PAL) to form trans-cinnamic acid [18]. The five distinct biosynthetic routes characterized for 5-CQA are shown in Figure 3, with Pathways 1 and 2 being the most prevalent in plants, having been confirmed in widely studied families such as *Asteraceae*, *Solanaceae*, and *Rubiaceae* [21]. In contrast, Pathways 3 and 4 represent specialized branches derived from enzymes unique to certain plant species. Notably, Pathway 5 operates independently of the others and has thus far been reported exclusively in sweet potato (*I. batatas*) [9,22,23].

5-CQA biosynthesis primarily occurs in the cytoplasm; However, synthesis has also been observed in chloroplasts of *L. japonica*, with final storage occurring in vacuoles [24]. In both Pathways 1 and 2, trans-cinnamic acid is converted into *p*-coumaroyl-CoA through the sequential action of cinnamate 4-hydroxylase (C4H) and 4-coumarate: CoA ligase (4CL). Subsequently, Pathways 1 and 2 diverge through the actions of hydroxycinnamoyl-CoA:shikimic acid/quinic acid hydroxycinnamoyltransferase (HCT) and hydroxycinnamoyl-CoA:quinic acid hydroxycinnamoyltransferase (HQT). Pathway 1 involves HCT-catalyzed transesterification of shikimate with Acyl-CoA to form *p*-coumaroyl shikimate. In contrast, Pathway 2 utilizes HQT to catalyze transesterification of quinate with Acyl-CoA, yielding *p*-coumaroyl quinate [25]. In Pathway 2, *p*-coumaroyl quinate is directly hydroxylated by *p*-coumaroyl-shikimic acid/quinic acid 3-hydroxylase (C3’H) to produce 5-CQA. In Pathway 1, *p*-coumaroyl shikimate is first hydroxylated by C3’H to caffeoyl shikimate. HCT then transfers the acyl group to form caffeoyl-CoA, followed by HQT-mediated conjugation with quinate to yield 5-CQA. HCT and HQT are structurally similar enzymes that catalyze reversible acyl-transfer reactions between Acyl-CoA and their respective substrates, and HCT exhibits substrate preference for shikimate, whereas HQT favors quinate [25]. Pathway 3 involves the direct hydroxylation of *p*-coumaric acid to caffeic acid, catalyzed by coumarate 3-hydroxylase (C3H). The resulting caffeic acid is then activated to caffeoyl-CoA by 4CL, after which this pathway converges with Pathway 1 [9,26]. Pathway 4 diverges from Pathway 1 at caffeoyl shikimate, which is hydrolyzed by caffeoyl shikimate esterase (CSE) to form caffeic acid, after which it rejoins Pathway 3 [27]. While seemingly redundant for 5-CQA synthesis, this pathway redirects metabolic flux away from H-lignin toward G/S-lignins in *A. thaliana* [27]. Similar redirection occurs in *Panicum virgatum* L., *Populus deltoides* Marshall, *Medicago truncatula* Gaertn., and the gymnosperm *Larix kaempferi* (Lamb.) Carrière [28,29,30]. Notably, CSE homologs are absent in *B. distachyon* and *Zea mays* L. [30]. Pathway 5 appears to be unique to *I. batatas* [22,23]. In its roots, UDP-glucose:cinnamic acid glucosyltransferase (UGCT) first glucosylates trans-cinnamic acid to form cinnamoyl glucose. After hydroxylation at positions 3 and 4 of the aromatic ring to yield caffeoyl glucose, HCGQT transfers the caffeoyl moiety to quinate, synthesizing 5-CQA [9,31].

Beyond pathway 5, which is unique to sweet potatoes, the gene expression profiles of other 5-CQA biosynthetic pathways exhibit significant variation across plant taxa [27]. Substantial evidence indicates that Pathway 4, involving CSE, and the preceding Pathway 1, serve as the primary routes for lignin biosynthesis in plants. Although CSE is absent in some species, this does not negate its crucial role in the conversion of caffeoyl shikimate in numerous plants, including eudicots like *Salix psammophila*, *P. virgatum*, *P. deltoides*, *Petunia x hybrida* and *Medicago truncatula*, as well as the gymnosperm *L. kaempferi* [28,29,30,32,33,34]. Pathway 1 generates caffeoyl-CoA via a reverse reaction of HCT, whereas Pathway 4 bypasses this step through the coordinated actions of CSE and 4CL. Given the central role of CSE in lignin biosynthesis, it can be inferred that Pathway 4 likely represents the major route for 5-CQA synthesis in most plants. Conversely, species lacking CSE (e.g., *B. distachyon* and *Z. mays*) likely employ alternative pathways. Pathway 2, catalyzed by C3’H and HQT from *p*-coumaroyl-CoA, offers the most direct route. However, as this pathway does not align with the metabolic flux of 5-CQA biosynthesis, it is probably only a branch for 5-CQA synthesis and not considered a major route. In Pathway 3, the bifunctional peroxidase C3H, characterized in *Arabidopsis* and *B. distachyon*, can directly convert coumaric acid to caffeic acid [28]. This discovery has led to a revision of the traditional lignin biosynthesis model. Although subsequent research on C3H remains limited, it is plausible that plants lacking CSE synthesize 5-CQA primarily through a combination of Pathways 1 and 3.

The prevailing view is that the conversion of 5-CQA to its isomers, 3-CQA and 4-CQA, occurs through a non-enzymatic mechanism. In an alkaline environment, 5-CQA undergoes a spontaneous acyl migration to form these isomers [35]. This is supported by the in vitro enzymatic assays of *HCT* in tea (*Camellia sinensis* (L.) Kuntze) conducted by Chen et al., who observed that as the pH of the reaction solution increased from 4 to 10, the level of 5-CQA decreased, while the levels of 3-CQA and 4-CQA correspondingly increased [36].

In contrast to the spontaneous acyl migration forming 3-CQA and 4-CQA, the biosynthesis of diCQAs and triCQAs is now increasingly understood to be enzyme-catalyzed. While diCQAs and triCQAs are widely distributed in plants, their biosynthetic mechanisms remain poorly characterized. Moglia et al. demonstrated that in tomato (*Solanum lycopersicum* L.), HQT not only synthesizes 5-CQA but also catalyzes the condensation of two 5-CQA molecules (acting as acyl acceptor and donor, respectively) to form 3,5-diCQA and quinate. Through heterologous expression in *Escherichia coli*, they confirmed HQT in vitro capacity for diCQA biosynthesis. The study further proposed compartment-specific HQT functions: vacuolar conditions—characterized by elevated 5-CQA concentrations and lower pH—favor diCQA synthesis [37]. A recent study on sweet potato (*I. batatas*) revealed that HCT also exhibits dual functionality [38]. However, in vitro assays showed HCT generated only trace amounts of diCQA, insufficient to account for the high diCQA levels observed in planta. Consequently, the researchers identified IbICS, a GDSL hydrolase that similarly catalyzes 3,5-diCQA formation from two 5-CQA substrates, as shown in Figure 3. Notably, IbICS exhibits significantly higher catalytic efficiency than HCT. Its stable expression in Pichia pastoris offers a promising platform for industrial diCQA production.

### 3.2. Structural Genes Involved

The biosynthesis of 5-CQA is regulated by multiple structural genes. These genes directly encode functional enzymes in the 5-CQA pathway, thereby governing 5-CQA production [9,18,39]. As previously established, CQAs biosynthesis occurs through the phenylpropanoid pathway [18]. This pathway shares close connections with lignin and flavonoid biosynthesis [39].

Phenylalanine ammonia-lyase (PAL), a pivotal enzyme in the plant phenylpropanoid pathway, catalyzes the deamination of phenylalanine to trans-cinnamic acid. This reaction initiates the phenylpropanoid pathway, bridging primary and secondary metabolism [40]. In rice (*Oryza sativa* L.), PAL-generated trans-cinnamic acid serves as the precursor for flavonoids, lignins, salicylic acid, and other bioactive compounds. *PAL* silencing reduces insect resistance [41]. Tobacco (*Nicotiana tabacum* L.) with silenced *PALs* shows decreased5-CQA and other phenolic compounds, correlating with diminished disease resistance [42]. Furthermore, overexpression of cloned *AmPAL* from *Astragalus membranaceus* (Fisch.) Bunge in *Nicotiana benthamiana* significantly enhanced saline-alkaline soil adaptation compared to wild-type plants [43].

Cinnamate 4-hydroxylase (C4H), a cytochrome P450 monooxygenase (CYP450), catalyzes the second committed step in the phenylpropanoid pathway [44]. This enzyme mediates the hydroxylation of trans-cinnamic acid to *p*-coumaric acid. As *p*-coumaric acid serves as a precursor for diverse secondary metabolites, alterations in *C4H* expression directly modulate flavonoid and lignin biosynthesis, consequently impacting stress resilience [45]. In tea plant (*C. sinensis*), *C4H* transcription responds to environmental stressors. Under hormonal, cold, drought, and high-salinity conditions, *CsPAL*, *CsC4H*, and *Cs4CL* genes exhibit tissue-specific expression patterns [44].

4-Coumarate: CoA ligase (4CL) functions as a critical branch point in the phenylpropanoid pathway. This enzyme activates diverse hydroxylated and methoxylated phenylpropanoid derivatives to their corresponding CoA thioesters, including caffeoyl-CoA, *p*-coumaroyl-CoA, and feruloyl-CoA [46]. In ‘Crown’ plum (*Prunus salicina* Lindl.), genome-wide analysis identified eight *Ps4CL* genes. Among these, *Ps4CL1*, *Ps4CL2*, *Ps4CL7*, and *Ps4CL8* likely regulate lignin biosynthesis in fruit mesocarp, with all family members containing hormone-responsive cis-elements [47]. Transgenic tobacco expressing a *Populus tomentosa* Carr. fusion gene *4CL1-CCR* exhibited 18.7% higher total G + S lignin content in stems compared to wild-type controls. Notably, S-lignin levels remained unchanged due to unaltered expression of *F5H*, which mediates G-to-S lignin conversion [48].

In 2019, Jaime Barros et al. identified *p*-coumaric acid 3-hydroxylase (C3H) as a bifunctional peroxidase that directly hydroxylates p-coumarate to caffeate. The corresponding gene was cloned in *B. distachyon* and *A. thaliana* [26]. Their work established C3H as the only non-membrane-bound hydroxylase in lignin biosynthesis, fundamentally revising accepted phenylpropanoid pathway models [26]. Subsequent *C3H* knockout in *B. distachyon* by Shrestha et al. significantly altered phenylpropanoid enzyme profiles in stems and disrupted cellular redox homeostasis [49].

Coumaroyl shikimate/quinate 3′-hydroxylase (C3’H) belongs to the cytochrome P450 CYP98 family. As a key rate-limiting enzyme in the phenylpropanoid pathway, CYP98 cooperates with HCT to metabolize *p*-coumaroyl-CoA—the central precursor for CQAs and flavonoid biosynthesis [39]. *C3’H* knockout in the *Physcomitrium patens* significantly reduced transcription of both upstream and downstream structural genes, ultimately diminishing phenylpropanoid end-product accumulation [50]. It is crucial to distinguish between C3H and C3’H, as they are distinct enzymes with different functions and substrate specificities, playing fundamentally different roles in the phenylpropanoid pathway, and the two should not be conflated.

Caffeoyl shikimate esterase (CSE) was first characterized in 2013, leading to a fundamental revision of established lignin biosynthesis model [27]. In *P. x hybrida* hort. ex Vilm. ‘Mitchell diploid’ (MD), Kim et al. used RNAi-mediated CSE down-regulated (*ir-PhCSE*) Petunias. These transgenics exhibited reduced stem lignification and growth impairment. And many floral volatiles benzenoid/phenylpropanoid (FVBP) genes were influenced, including elevated transcripts *C3H*, *HCT* and *4CL*, alongside significantly decreased phenylalanine and caffeate pools [51]. In *L. kaempferi*, transcriptome-guided cloning identified LkCSE with confirmed catalytic function. This enzyme exhibits strict tissue-specific expression, showing 50-fold higher transcript levels in stems than roots. This represents the first functional characterization of CSE in gymnosperms [30].

Both HCT and HQT belong to the BAHD acyltransferase family, sharing two conserved motifs: “HXXXD” and “DFGWG” [52]. Their substrate specificities differ: HCT preferentially utilizes shikimate, whereas HQT exclusively accepts quinate. Both enzymes activate *p*-coumaroyl-CoA to form *p*-coumaroyl quinate (via the “quinate shunt”) or *p*-coumaroyl shikimate (via the “shikimate shunt”) [9]. Crucially, these enzymes catalyze reversible reactions. For example, in the quinate shunt, HCT regenerates caffeoyl-CoA from caffeoyl shikimate, which HQT then conjugates with quinate to form 5-CQA. Liu et al. [53] cloned *TaHQT1* and *TaHQT2* from *Taraxacum antungense* Kitag., with *TaHQT1* showing root-predominant expression and *TaHQT2* leaf-predominant expression. Knockout lines showed reduced 5-CQA accumulation, while heterologous expression enhanced production, confirming both isoforms’ essential roles in *T. antungense* 5-CQA biosynthesis.

The inter-relationships among the key genes (*PAL*, *C4H*, *4CL*, *HCT*, *HQT*, etc.), their encoded enzymes, and associated metabolites are summarized in Figure 3, which depicts the integrated biosynthetic and regulatory network leading to CQAs formation.

### 3.3. Transcriptional Regulation

**Figure 4:**
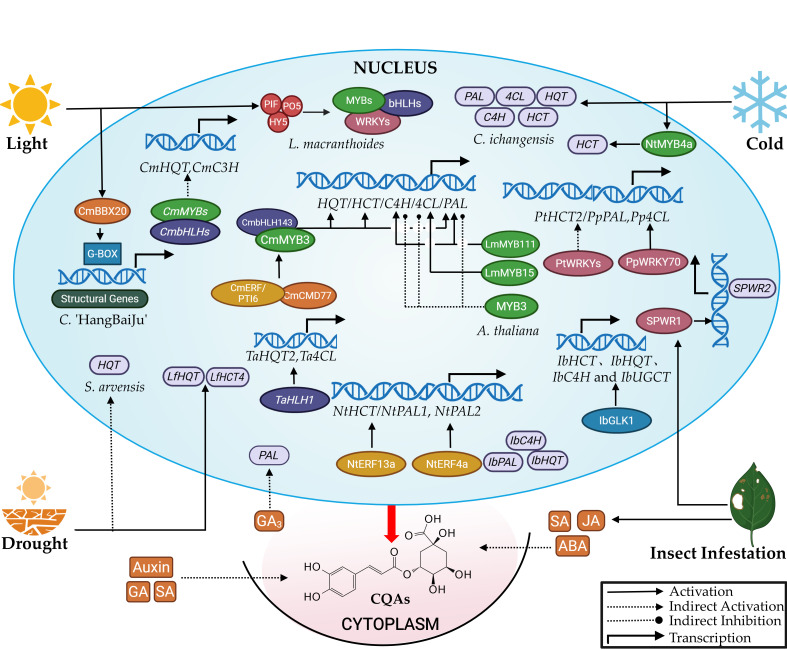
Regulation of CQAs biosynthesis.

Transcription factors (TFs) regulate CQAs accumulation by controlling structural gene expression [11,54]. These TFs bind specifically to promoter regions of target genes, activating or repressing their transcription to modulate CQAs levels [11]. The characterization of numerous transcription factor (TF) regulators in the CQAs biosynthesis pathway is summarized in Figure 4.

The MYB transcription factor family represents one of the most extensively studied TF groups in plants, playing pivotal roles in growth regulation, secondary metabolism, signal transduction, and biotic/abiotic stress responses. These TFs contain a conserved DNA-binding domain, MYB domain, typically featuring 1–4 tandem imperfect repeats (Rs) at the N-terminus. Based on Rs number and arrangement, MYB TFs are classified into four subfamilies [55]. In *C. morifolium* ‘HangBaiJu’, Lu et al. [56] identified *CmERF/PTI6* and *CmCMD77* as upstream modulators of *CmMYB3* and *CmbHLH143* through integrated metabolomics and transcriptomics. CmMYB3 potentially forms complexes with CmbHLH143 to directly activate structural genes *CmPAL1/2*, *CmCHS1/2*, *CmFNS*, *CmHQT* and *CmHCT*, thereby coordinating flavonoid and CQAs biosynthesis. In *Lonicera macranthoides*, Chen et al. identified an R2R3 MYB transcription factor, LmMYB111, through integrated transcriptomic and metabolomic analysis [11]. They demonstrated that Overexpression of *LmMYB111* in both tobacco and honeysuckle resulted in a significant increase in the content of CQAs and luteoloside. Their RNA-seq analysis revealed that this overexpression markedly upregulated the expression of several structural genes, including 10 *PALs*, 3 *C4Hs*, 7 *4CLs*, and 4 *HCT/HQTs*. Furthermore, through electrophoretic mobility shift assay (EMSA) and dual-luciferase reporter (DLR) assays, they provided evidence that LmMYB111 specifically binds to the promoter regions of LmMYB4, *LmPAL1*, *Lm4CL2*, *LmCHI*, and *LmDFR*, thereby promoting the excessive accumulation of CQAs, luteoloside, and other phenolic compounds. Previously, this research team confirmed that another R2R3 MYB transcription factor in honeysuckle, *LmMYB15*, directly binds to the promoter of the key gene *4CL*, enhancing CQAs accumulation and boosting phenylpropanoid pathway activity [57]. Luo et al. overexpressed the tobacco-derived *NtMYB4a* TF. *NtMYB4a* overexpression upregulated *NtPAL* and *Nt4CL*, while knockout downregulated these genes [58]. Interestingly, the nucleus-localized transcription factor MYB3 in *A. thaliana* has been reported to suppress the excessive accumulation of lignin and anthocyanins under high-salinity stress. When subjected to high salt conditions, *myb3* mutant plants accumulated significantly higher levels of lignin and anthocyanins compared to wild-type controls. The expression of related biosynthetic genes, such as *PAL1*, *C4H*, and *4CL*, was also elevated. These findings indicate that MYB3 acts as a transcriptional repressor under high-salinity stress, downregulating genes associated with the phenylpropanoid pathway, including those involved in CQAs biosynthesis [59]. Guan et al. conducted multi-omics analysis across flowering stages in honeysuckle (*L. japonica*). MYB, AP2/ERF, and NAC TF families were shown to positively regulate CQAs biosynthesis [60].

The WRKY transcription factor family mediates plant responses to drought, cold, heat, and salinity stresses while regulating signaling networks and structural genes in CQAs biosynthesis [61,62]. Transient overexpression of *PtWRKY60*, *PtWRKY89*, *PtWRKY93*, *PtWRKY38*, and *PtWRKY45* in poplar (*P.tomentosa*) significantly increased expression levels of *PtHCT2*, correspondingly elevating 5-CQA accumulation [63]. Wang et al. cloned *NtWRKY41a* from tobacco, demonstrating that its overexpression enhances CGA and anthocyanin biosynthesis while suppressing scopolin and flavonoid production. Mechanistically, NtWRKY41a activates *NtCCoAOMT* and *NtHST* promoters while repressing *NtF6’H1* and *NtGT3* transcription [64] (Figure 3). In peach (*Prunus persica* (L.) Batsch), PpWRKY70 binds and activates *PpPAL* and *Pp4CL* promoters. Under methyl jasmonate or pathogen induction, this increases total phenolics, flavonoids, and CGA accumulation in fruit [65].

The basic helix-loop-helices (bHLHs) proteins represent one of the largest families of transcription factors found in plants [66]. In *T. antungense*, the transcription factor TaHLH1 enhances 5-CQA and Cynaroside biosynthesis by directly binding promoters of *TaHQT2* and *Ta4CL*. *TaHLH1*-overexpressing lines showed increases 5-CQA accumulation, while RNAi-silenced lines exhibited reduction, confirming its regulatory role [67]. Zhao et al. revealed why octoploid *Chrysanthemum* ‘Gongju’ accumulates more CQAs than tetraploid counterparts through integrated transcriptomics and metabolomics. Elevated *CmHQT* and *CmC3H* expression drives enhanced CGA production in octoploids. Co-expression networks identified *CmMYB* and *CmbHLH* as key regulators of *CmHQT* and *CmC3H* [68]. *Vaccinium dunalianum* Wight shows developmentally regulated CQAs accumulation, Leaf CQAs accumulation varied significantly across developmental stages. Zhang et al. identified 15 core structural genes: four *PAL*, two *C4H*, four *4CL*, two *HCT*, and three *C3H* homologs—the first comprehensive characterization in this species. Three putative regulators were also discovered: two *bHLH* and one *WRKY* transcription factors. This provides novel insights into CQAs regulatory networks [69].

The GARP (Golden2, ARR-B, Psr1) family constitutes a group of plant-specific transcription factors that regulate a diverse array of physiological processes [70]. Luo et al. profiled CQAs across 16 sweet potatoes (*I. batatas*) genotypes, establishing correlations between CQAs accumulation and antioxidant capacity, and Comparative transcriptomics identified *IbGLK1*, a GARP-family TF. Yeast one-hybrid and dual-luciferase assays confirmed *IbGLK1* directly activates promoters of *IbHCT*, *IbHQT*, *IbC4H*, and *IbUGCT*, thereby enhancing CQAs biosynthesis [11].

AP2/ERF transcription factors play multifaceted roles in regulating the biosynthesis of various specialized metabolites in response to diverse environmental stresses [71]. Wang et al. identified the *NtERF* gene, a member of the ERF family’s IXa subfamily, within the tobacco genome [71]. Their research demonstrated that NtERF13a specifically binds to GCC-box or DRE element segments in the promoters of the *NtHCT*, *NtF3’H*, and *NtANS* genes [72]. This binding activates their transcription and promotes the biosynthesis of CQAs, flavonoids, and lignin, this regulatory model is summarized in Figure 4. Furthermore, He et al. identified two *NtERF* genes involved in the biosynthesis of CQAs and flavonoids in tobacco. Overexpression of *NtERF4a* significantly enhanced the accumulation of CQAs and flavonoids in tobacco leaves, whereas its silencing had the opposite effect. Chromatin immunoprecipitation and dual-luciferase reporter assays demonstrated that NtERF4a directly binds to the GCC-box element in the promoters of *NtPAL1* and *NtPAL2*, thereby activating their expression.

### 3.4. Hormonal Regulation

Phytohormones serve as essential endogenous signaling molecules within plant transduction networks, modulating both developmental processes and stress responses, as overviewed in Figure 4 [73]. Salicylic acid (SA)—critically associated with biotic/abiotic stress resistance—demonstrates reciprocal interaction with CQAs. Studies in *I. batatas* and *Z. mays* indicate CQAs-mediated SA pathway activation enhances stress tolerance [74,75]. Notably in *I. batatas*, exogenous application of GA, SA, or ABA to shoot apices consistently elevated CQAs accumulation. Hormone-responsive cis-elements were identified in promoters of multiple CGA biosynthetic genes, confirming tight hormonal coordination of this pathway [76].

Gibberellins (GAs) serve as crucial regulators of plant growth and are increasingly recognized as modulators of CQAs biosynthesis. In *Echinacea purpurea* (L.) Moench, GA3 application at varying concentrations differentially influenced hairy root growth and metabolite profiles. Optimal GA3 concentrations significantly enhanced 5-CQA accumulation while upregulating *PAL* gene expression [77]. Similarly in *Fagopyrum esculentum* Moench, combined GA and IAA treatments maximized production of 5-CQA, caffeic acid, and flavonoids [78]. Sharaf et al. examined GA3 effects on CQAs distribution in *Cynara scolymus* L., applying treatments to leaves and monitoring receptacle tissues. Transplanted seedlings with controlled development showed substantially increased foliar CQAs following GA3 application. However, receptacle CQA consistently decreased across treatments. GA3 likely redirects phenolic allocation in artichoke, reducing CQA accumulation in receptacles [79].

The role of auxins (IAA) in plant growth regulation is well-established [80]. Emerging evidence indicates auxins significantly influence CQAs biosynthesis. In *C. morifolium*, Ghimire et al. observed concentration-dependent increases in CQAs accumulation following NAA and IAA treatments, though IBA showed no significant effect [81]. Wang et al. overexpressed *MdIAA24*, a key auxin biosynthesis gene, in *Malus domestica* Borkh. Following *Glomerella leaf spot* pathogen infection, these transgenics exhibited substantially enhanced CQAs accumulation compared to wild-type plants [82].

### 3.5. Environmental Regulation

Temperature, light, drought, and herbivory differentially regulate CQAs biosynthesis in plants. Under suboptimal growth conditions, plants activate physiological adaptations to mitigate environmental stressors. Secondary metabolites including CQAs and flavonoids serve as critical components of these adaptive metabolic responses [83]. Their specific roles and relationships within this network are summarized in Figure 4.

Light serves dual roles in plants: as the primary energy source for growth and as a key developmental signaling cue. Variations in light intensity, spectral quality, and photoperiod significantly modulate secondary metabolite biosynthesis [84]. In *chrysanthemum* ‘HangBaiJu’, Lu et al. employed integrated transcriptomic and metabolomic analyses to identify key regulators of flavonoid and CQAs biosynthesis, namely CmMYB3/6/16 and CmBBX20/22.2/22.3. Subsequent transient overexpression assays and Y1H experiments confirmed that CmBBX20 directly binds to the G-Box cis-element in the promoters of downstream target genes, thereby promoting the accumulation of flavonoids and CQAs [85]. Reduced light intensity in *Marsdenia tenacissima* (Roxb.) Moon suppresses CQAs biosynthesis and downregulates associated pathway genes [86]. Similar light-dependency occurs in *L. japonica*, where shading treatments substantially decrease foliar 5-CQA accumulation. A co-expression network integrates 5-CQA biosynthetic genes with carbohydrate metabolism, photosynthetic components, light-signaling elements, and transcriptional regulators [87]. Beyond intensity, light spectral quality profoundly influences 5-CQA biosynthesis across species. Blue light upregulates *FvHCT* expression and enhances 5-CQA production in strawberry (*Fragaria × ananassa* (Weston) Duchesne ex Rozier). Different light spectra exert multifaceted physiological effects in strawberry fruits [88]. Continuous blue light combined with elevated CO_2_ increases 5-CQA accumulation in lettuce (*Lactuca sativa* L.) by activating *PAL* and downstream enzymes [89]. Blue light coupled with low temperatures maximizes 5-CQA production in *Berula erecta* (Huds.) Coville [90]. During potato tubers (*Solanum tuberosum* L.) storage, sodium-vapor and fluorescent lighting enhance 5-CQA accumulation more effectively than high-pressure mercury lamps. Light exposure also induces metabolic conversion of 5-CQA to 3-CQA or 4-CQA isomers [91].

Cold stress significantly impacts plant growth and development [92]. In *Citrus ichangensis* Swingle, cold exposure upregulates *PAL*, *C4H*, *4CL*, and *HCT/HQT* expression, enhancing 5-CQA accumulation. This cold-induced CGA confers frost tolerance through ROS scavenging [15]. Xie et al. identified *NtMYB4a* as a cold-responsive transcription factor regulating CQAs biosynthesis in tobacco. At ambient temperatures, NtMYB4a-overexpressing and wild-type lines showed comparable CQAs levels. Following cold treatment, transgenics exhibited substantially elevated CQAs accumulation, *4CL*/*HCT* expression, and enzyme activity versus wild type. *p*-Coumaroyl quinate levels remained similar in wild-type and knockout lines at normal temperatures. Cold exposure increased *p*-coumaroyl quinate in wild-type but decreased it in knockouts. Overexpressors maintained elevated *p*-coumaroyl quinate across all conditions. *NtMYB4a* activates *HCT* transcription under cold stress, enhancing *p*-coumaroyl quinate production to boost CQAs biosynthesis [93]. Sim et al. examined phenolic profiles in *Aster × chusanensis* Ling cultivated at varying temperatures. Optimal elevated temperatures maximized foliar CQAs accumulation, the dominant phenolic acid [94]. Goławska et al. compared cultivated and wild *Viburnum dilatatum* Thunb., revealing temperature-dependent variations in phenolic/flavonoid profiles. Thermal regimes profoundly influence CQAs biosynthesis across ecotypes [95].

Drought stress is a climatic phenomenon characterized by seasonal occurrence that severely affects crop yields [96]. In field sowthistle (*Sonchus arvensis* L.), drought stress notably induced the upregulation of genes involved in the CQAs biosynthesis pathway. Under conditions of 50% field capacity, the concentrations of chicoric acid, 5-CQA, and 2,5-dihydroxybenzoic acid reached their highest levels. A strong association was observed between HQT expression and 5-CQA content, indicating that the HQT-dependent mediated pathway serves as the primary route for CQAs in *S. arvensis* [97]. Khan et al. observed divergent CGA responses in rice (*O. sativa*) under drought: cultivar PR-115 showed markedly enhanced accumulation, while Super-7 exhibited substantial reduction [98]. *Amaranthus* L. progressively increased phenolic acids including CQAs with intensifying drought severity [99]. Contrastingly, drought reduced both CQAs and flavonoids in *Ligularia fischeri* (Ledeb.) Turcz. Downregulation of *LfHCT* and *LfHQT4* directly diminished CQA production, consistent with *HCTs* established role in *Asteraceae* CQAs biosynthesis [100]. Ghafari et al. applied partial root-zone drying (PRD) to *M. domestica*, comparing alternating (APRD) and fixed (FPRD) drying regimes. At 75% crop evapotranspiration (ETc), both PRD methods substantially elevated 5-CQA, caffeate, and catechin accumulation without compromising yield. PRD likely enhances water-use efficiency by activating antioxidant systems, hormone signaling, and drought-responsive transcription factors [101].

Insect infestations severely impact crop yields. Recent studies have reported that insect herbivory can induce the accumulation of CQAs [75,102,103]. In rice (*O. sativa*), the white-backed planthopper (WBPH, *Sogatella furcifera* (Horváth, 1899)) is a major migratory pest. Xie et al. conducted integrated transcriptomic and metabolomic analyses of a susceptible cultivar (TN1) and a resistant cultivar (KL35). They identified CQAs as key compounds in rice resistance to WBPH, with significantly higher levels in KL35 than in TN1, both before and after WBPH infestation. The study also pinpointed two critical resistance-related genes, *4CH* and *C4L*, hypothesizing that they are key genes in the CQAs biosynthetic pathway of the highly resistant cultivar KL35 [102]. In sweet potato, the sweetpotato weevil (SPW, *Cylas formicarius* (Fabricius, 1798)) is one of the most destructive pests. Hou et al. observed that SPW feeding induced the accumulation of CQAs in sweet potato leaves. By investigating the associated hormonal networks and key genes, they demonstrated that SPW herbivory stimulates the production of jasmonic acid, salicylic acid, and abscisic acid. These hormones upregulate key CQA biosynthetic genes, including *IbPAL*, *IbC4H*, and *IbHQT*, thereby enhancing CQAs synthesis. Subsequently, the same team identified two major SPW resistance genes, *SPWR1* and *SPWR2*, from two SPW-resistant sweet potato germplasms. Their research showed that SPW feeding induces the WRKY transcription factor SPWR1, which directly activates the expression of SPWR2. SPWR2, in turn, promotes the accumulation of CQAs and other resistance-related compounds in sweet potato [75,103].

### 3.6. Metabolic Engineering via Synthetic Biology Strategies

The elucidation of the CQAs biosynthetic pathway has laid a foundation for employing synthetic biology strategies to reconstruct and optimize this pathway in microbial chassis, aiming for the sustainable and scalable production of these high-value compounds. Recent studies have demonstrated significant progress in engineering efficient cellular factories, particularly in *E. coli* and *Saccharomyces cerevisiae*. In *E. coli*, Wang et al. established a de novo pathway from simple carbon sources to CQAs [104]. By crucially enhancing the activities of the cofactor-regenerating enzymes quinate/shikimate 5-dehydrogenase (YdiB) and 4-hydroxyphenylacetate 3-monooxygenase (HpaBC), they achieved a high CQAs titer of 2789.2 mg/L in a 5 L bioreactor. Similarly, Zhang et al. integrated a tyrosine ammonia-lyase (TAL) from *Rhodococcus taiwanensis* and implemented a multi-level metabolic engineering strategy, constructing the engineered strain CGA13 capable of producing CQAs solely from glucose, with a final titer of 1.53 g/L [105]. To further improve pathway efficiency, Hu et al. applied semi-rational design to the key acyltransferase HQT, significantly enhancing its selectivity for caffeoyl-CoA and *p*-coumaroyl-CoA, thereby effectively reducing byproduct formation [106]. In the eukaryotic chassis *S. cerevisiae*, consecutive studies by Zhong et al. realized de novo CQAs synthesis [107,108]. Their strategy involved introducing a TAL from *Rhodotorula glutinis* (RgTAL), deleting *HAM1* and *YJL028W* to elevate intracellular pools of the precursor coumaric acid, and employing fusion expression of *AtC4H* and *At4CL1* alongside co-expression of *CsHQT* and *AtC3’H*. Furthermore, they optimized the P450 enzyme microenvironment by overexpressing the lipid biosynthesis transcription factor *INO2* and deleting the heme oxygenase gene *HMX1*. Through comprehensive fermentation optimization, the CQAs titer was dramatically increased from 234.8 mg/L to 837.2 mg/L in shake flasks. These successful cases signify a transition from proof-of-concept to process development for the synthetic biological production of CQAs, highlighting the immense potential of systematic pathway design, enzyme engineering, and host metabolic network remodeling to achieve efficient and sustainable bio-manufacturing.

## 4. Bioactivity of CQAs

### 4.1. In Pharmacological

Compelling evidence from numerous studies demonstrates that CQAs exhibits a broad spectrum of pharmacological activities. These include anti-inflammatory, antimicrobial, antiviral, and anticancer effects, as well as protective roles in the nervous and cardiovascular systems, antidiabetic properties, and hepato-renal protection. Consistent findings indicate that CQAs are a class of polyphenolic compounds with multi-faceted bioactivity. These diverse and potent pharmacological properties have garnered significant and growing research interest in recent years.

**Figure 5:**
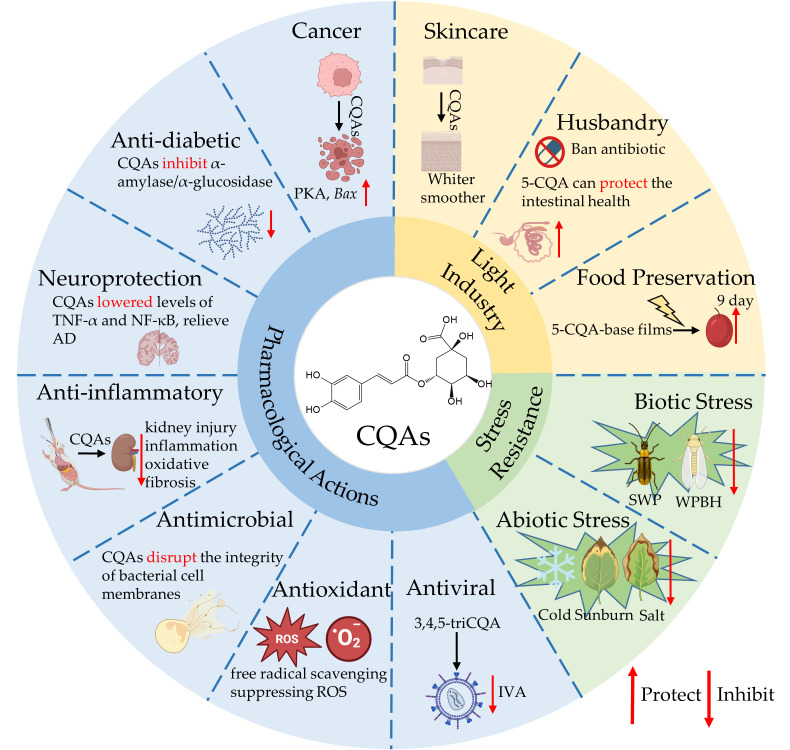
Bioactivity of CQAs. Abbreviations: PKA, protein kinase A; *Bax*, *Bcl-2-associated x*; TNF-α tumor necrosis factor-alpha; NF-κB, nuclear factor kappa-light-chain-enhancer of activated B cells; AD, Alzheimer’s disease; ROS reactive oxygen species; IAV, influenza A virus; WPBH, white-backed planthopper; SWP, sweet potato weevil; ^•^O_2_^−^ Superoxide anion.

Regarding anti-inflammatory effects, Lee et al. demonstrated that administering 5-CQA to STZ-induced diabetic rats significantly reduced cytokine levels, CRP, and CYP1A enzyme activity in a dose-dependent manner. This suggests a potential therapeutic strategy for managing inflammation in diabetes [109]. In an LPS-induced immune stress model in broilers, Tan et al. found that dietary supplementation with 5-CQA alleviated intestinal barrier damage and inflammation. The mechanism involved suppressing pro-inflammatory cytokines, TNF-α and IL-1β, promoting the anti-inflammatory cytokine IL-10, improving intestinal villus morphology, and enhancing the expression of the tight junction protein Claudin-2 in the ileum. Consequently, this led to improved growth performance [110]. In their study of LPS-induced inflammation and oxidative stress in RAW264.7 cells, Han et al. demonstrated that the isomers 5-CQA, 3-CQA, and 4-CQA entered cells via transmembrane transport and differentially inhibited the AKR1B1 enzyme, with potency depending on their hydrogen-bonding sites. Among them, 3-CQA exhibited the strongest AKR1B1 inhibitory effect, which correlated with its superior suppression of pro-inflammatory cytokines [111]. This structure-activity relationship and underlying mechanism are delineated in Figure 5. Jiao et al. treated a mouse model of renal fibrosis with varying concentrations of 5-CQA. Their findings indicate that 5-CQA likely alleviates kidney injury, inflammation, oxidative stress, and fibrosis by modulating the TLR4/NF-κB signaling pathway. This treatment also suppressed the expression of inflammatory factors and oxidative stress in both in vivo and in vitro fibrotic models [112]. Similarly, Xu et al. confirmed in a mouse study that 5-CQA inhibits cellular inflammation by downregulating the expression of TLR4, p-p38, p-p65, and p-IκBα. The anti-inflammatory effect was negated by TLR4 overexpression, which increased inflammatory cytokine levels. This suggests 5-CQA has potential as an anti-inflammatory agent in RAW264.7 cells and allergic rhinitis by regulating the TLR4/MAPK/NF-κB pathway [113]. Wang et al. used a chronic stress model in rats to show that 5-CQA treatment reduced hepatic inflammatory cytokine levels and NF-κB pathway activation, while increasing Resolvin D1 (RvD1) in the serum and liver [114]. They further found that the therapeutic effect was blocked by the inhibitor WRW4, indicating that 5-CQA protective effects are mediated by upregulating RvD1 to suppress the NF-κB pathway. Their work highlights the potential treatment value of 5-CQA for chronic inflammatory diseases. A recent study elucidated the anti-inflammatory mechanism and protein target of dihydrocaffeic acid (DA), a metabolite of 5-CQA, in acute pneumonia. Experiments in in vivo and in vitro inflammatory models confirmed DA’s anti-inflammatory effects. Using a designed DA molecular probe (DA-P) and activity-based protein profiling (ABPP), the protein target was identified as transaldolase 1 (TALDO1). Further analysis proposed that DA interacts with TALDO1 to regulate the PERK-IκBα-NF-κB signaling pathway, thereby inhibiting inflammation [5].

Regarding antioxidant properties, Xu et al. esterified 5-CQA with C18 fatty acids of varying unsaturation (C18:0, C18:1, C18:2, C18:3). They found that the liposolubility of the resulting derivatives decreased as the fatty acid unsaturation increased. Notably, all derivatives exhibited excellent oxidative stability, indicating their potential as highly effective antioxidants in lipid-based food systems [115]. Cejas et al. investigated the role of cholesterol in the protective effect of 5-CQA on model plasma membranes and its antioxidant capacity against lipid peroxidation. Their results showed that 5-CQA delayed lipid peroxidation and preserved the integrity of lipid vesicles [6]. Caruso et al. measured the superoxide antioxidant ability of caffeic and CQAs in several tea-based beverages. Both the plant extracts and the pure compounds exhibited strong antioxidant activity, almost completely eliminating superoxide radicals. The study also elucidated their mechanisms: although caffeic acid and CQAs scavenge radicals differently, both ultimately produce hydrogen peroxide (H_2_O_2_) and an [X-acid–η–O_2_], X = caffeic, 5-CQA, in an acidic environment with sufficient superoxide [116]. Similarly, using bioassay-guided analysis, Gloria et al. identified 5-CQA and 1-CQA as a major component in Avocado (*Persea americana* Mill.) Fruit Peel, responsible for its significant bioactivities, including free radical scavenging, suppressing ROS and lipid peroxidation, inhibiting nitric oxide production, and modulating antioxidant enzyme activities [117]. Furthermore, their Density Functional Theory analysis suggests that the radical scavenging mechanism of 5-CQA likely involves electron transfer, followed by proton transfer processes, primarily occurring at the 3’OH and 4’OH. This mechanism is schematized in Figure 5.

In neuroprotection, Kang et al. demonstrated that 5-CQA exerts potent antioxidant and neuroprotective effects in a rat model of cerebral artery occlusion. They proposed that it prevents neuronal death after cerebral ischemia by maintaining the binding of thioredoxin (Trx) to ASK1. Under excessive glutamate conditions, 5-CQA mitigated neurotoxicity and reduced ischemia-induced damage by modulating Trx to influence the balance of anti- and pro-apoptotic proteins. In a separate study the same year, Kang et al. also found that 5-CQA protects neurons by regulating the PP2A subunit B. Together, these studies highlight the compound’s potential as a neuroprotective agent [7,118]. In a study using a mouse model of cognitive impairment, Mosalam et al. investigated the combination of the PDE4 inhibitor cilomilast (CILO) with 5-CQA as a potential therapy for Alzheimer’s disease. They discovered that the combination activated the cAMP/PKA/CREB/BDNF signaling pathway, reduced neuronal damage, and lowered levels of neuroinflammatory markers, including TNF-α and NF-κB, demonstrating superior efficacy to individual treatments [119]. This synergistic mechanism and the key findings are summarized in Figure 5. In the same year, Mirzaei et al. also reviewed the protective effects of coffee components, including CQAs, against various neurodegenerative diseases like AD. They noted that these compounds typically mediate neuroprotection by modulating key signaling pathways and inflammatory mediators, such as NF-κB, Nrf2, interleukins, and TNF-α [120]. Furthermore, a study showed that 5-CQA acts as a caloric restriction mimetic. In human neuroblastoma SH-SY5Y cells, it reversed oxidative stress induced by amyloid-β peptide, restored the activity of Na^+^, K^+^-ATPase and Ca^2+^-ATPase pumps, and inhibited the overactivation of acetylcholinesterase. This multi-target synergistic action underpins its neuroprotective effect, positioning 5-CQA as a potential candidate for AD intervention strategies [121].

In cancer therapy, Huang et al. discovered that 5-CQA promotes the expression of p21 by downregulating miR-17 through the SUMOylation of c-Myc. This mechanism induces cancer cell differentiation and attenuates their malignant behavior. There in vivo experiments confirmed that 5-CQA treatment halted tumor growth. Notably, even at very high doses, no toxicity was observed [122]. Furthermore, research revealed that 5-CQA significantly enhances β-lapachone-induced Protein Kinase A activation in cancer cells, leading to cell death [123]. This proposed synergistic mechanism is illustrated in Figure 5. Concurrently, 5-CQA compounds present in mulberry (*Morus alba* L.) extract were shown to selectively inhibit the growth of MCF-7 breast cancer cells and exhibit strong free radical scavenging activity against DPPH radicals [124]. Moreover, Wang et al. identified mitochondrial Acetyl-CoA acetyltransferase 1 (ACAT1) as a direct target of 5-CQA for inhibiting tumor cell growth. Their study elucidated that 5-CQA, via non-covalent binding, inhibits phosphorylation at the Y407 site of ACAT1. This disrupts its role in regulating tumor cell metabolism and suppresses cancer cell proliferation, providing a mechanistic foundation for developing natural targeted anticancer drugs and the clinical application of 5-CQA [125]. 5-CQA as a cancer differentiation inducer, downregulates PD-L1 expression in tumor cells by inhibiting the STAT1/IRF1 pathway. This enhances the efficacy of anti-PD-1 antibodies, promotes the infiltration of CD8^+^ T cells into the tumor microenvironment, and augments granzyme B-mediated cytotoxicity. This suggests a novel combinatorial strategy to overcome the low response rates to immune checkpoint inhibitors [2]. Similarly, studies report that 5-CQA and gallic acid can effectively bind to the critical Thr308 phosphorylation site of AKT1. This binding inhibits the proliferation, migration, and invasion of liver cancer cells and promotes apoptosis [126]. Meanwhile, 5-CQA, a key active compound in purple carrot extract (PCE), demonstrates a selective inhibitory effect on MDA-MB-231 triple-negative breast cancer cells. It upregulates the expression of pro-apoptotic genes like *Bcl-2-associated x*(*Bax*), revealing its specific suppressive action against breast cancer [127].

In a study on antimicrobial applications, Fu et al. developed supramolecular nan particles from berberine and 5-CQA [4]. They found that these nanoparticles exhibited significantly stronger inhibitory effects against *Staphylococcus aureus* and methicillin-resistant *S. aureus* than ampicillin, oxacillin, BBR, 5-CQA, or a simple mixture of BBR and 5-CQA. Furthermore, in a mouse model of wound infection, the nanoparticles markedly promoted wound healing. Furthermore, a study by Kang et al. demonstrated that the bactericidal effect of 5-CQA combined with ultraviolet-A (365 nm) was significantly greater than either treatment alone [128]. Their results indicated that the primary mechanism involves disruption of the bacterial cell membrane, leading to the leakage of intracellular contents and DNA degradation. The combination also reduced succinate-coenzyme Q reductase activity by approximately 72%, enhanced bacterial sensitivity to antibiotics, and inhibited the spread of drug resistance. Based on these findings from this research, we have summarized the proposed mechanism of action in Figure 5. Han et al. reported that 0.5 mg/mL 5-CQA acts as an effective antimicrobial agent against both *E. coli* and *S. aureus* when activated by light [129]. By incorporating 5-CQA as a photosensitive antibacterial agent into an agar film, they extended the shelf life of cherries by 9 days, demonstrating its potential for light-based preservation technologies. Similarly, Yang et al. successfully synthesized chitosan-grafted-chlorogenic acid (CS-g-CA) using a carbodiimide method. They confirmed that CS-g-CA significantly inhibits *S. aureus* growth, with a minimum inhibitory concentration of 0.625 mg/mL. The compound exerts its effects by disrupting cell membrane integrity, inhibiting antioxidant enzyme activity, interfering with DNA metabolism, and reducing extracellular polymeric substance production [130]. Beyond *S. aureus*, 5-CQA has demonstrated antimicrobial effects against other pathogens. For instance, Liu et al. found that a combined treatment of low-dose 5-CQA and *p*-coumaric acid drastically increased cell permeability and inhibited biofilm formation in *Shigella*. This synergistic action significantly inactivated Shigella and, when applied to fresh-cut tomatoes, showed efficacy comparable to chemical disinfectants, supporting the development of potent, low-dose natural preservatives [131]. In the context of COVID-19-associated mucormycosis (CAM), research indicates that 5-CQA and quercetin, derived from the ethanol extract of *Thymus vulgaris* subsp., showed high binding affinity for key CAM-related proteins, namely EGFR and GRP78. Using an integrated approach of HPLC, molecular docking, ADME analysis, and molecular dynamics simulations, the study proposed 5-CQA and quercetin as promising anti-CAM drug candidates. However, this conclusion requires further validation through in vitro and in vivo experiments to confirm their clinical safety and efficacy [132]. Jiang et al. also reported that mixing or spraying with a 5-CQA solution produces significant antimicrobial effects against *S. aureus*, *E. coli*, *Candida albicans*, and *Streptococcus pneumoniae* [133].

Regarding antiviral activity, Wang et al. isolated eight CQAs from *Ilex pubescens* Hook. & Arn leaf extract, all of which exhibited anti-influenza virus activity as neuraminidase (NA) inhibitors [3]. The most potent compound, 3,4,5-triCQA, demonstrated significantly higher inhibition than diCQAs. Its binding site on NA is suggested to be distinct from existing drugs like oseltamivir, marking it as a promising lead for a new anti-influenza class [3]. In a subsequent study, the same group delved into its cellular mechanism, revealing that 3,4,5-triCQA suppresses influenza A virus (IAV)-induced inflammation in host cells through the TLR3/7 signaling pathway [134]. This specific anti-inflammatory mechanism is schematized in Figure 5. Yang et al. reported that 5-CQA inhibits the proliferation of duck enteritis virus (DEV) in duck embryo fibroblast (DEF) cells by modulating the NF-κB signaling pathway. This finding suggests a potential strategy for preventing DEV infection and mitigating the associated economic losses in poultry production [135]. Against porcine deltacoronavirus (PDCoV), 5-CQA effectively suppressed viral self-replication, reducing the synthesis of viral RNA and proteins. It also significantly diminished apoptosis in PDCoV-infected cells and inhibited the release of a progeny virus. As a potent anti-PDCoV agent, 5-CQA provides a valuable reference for the development of related therapeutics [136].

In anti-diabetic research, studies utilizing rat models have demonstrated that 5-CQA effectively manages diabetes and its complications. As summarized in Figure 5, The proposed mechanisms include the inhibition of α-amylase/α-glucosidase, activation of the AMPK pathway, improvement of lipid metabolism, and exertion of antioxidant effects [137]. Furthermore, a recent multidisciplinary study integrating enzyme kinetics, spectroscopy, molecular simulations, and cell models elucidated the mechanism of action. It confirmed that 5-CQA binds to the active pocket of α-glucosidase through a mixed inhibition mechanism involving hydrogen bonding and hydrophobic interactions. This binding induces a conformational change in the enzyme, leading to its inactivation. Using the human liver cell line HepG2 to model glucose metabolism, the study also confirmed that 5-CQA significantly promotes glucose consumption at non-cytotoxic concentrations [138]. Additionally, the flower extract of *Sambucus nigra* L., rich in phenolic acids such as 5-CQA, has been investigated. Extracts from various elder cultivars demonstrated significant inhibition of α-glucosidase. A strong correlation was observed between high 5-CQA content and the extract’s potent enzyme inhibitory and antioxidant activities, which are crucial for its glucose-regulating effects. This finding provides a valuable reference for developing natural anti-diabetic therapeutics [139]. Similarly, in studies on *Cirsium setosum* (Willd.) MB., Zhou et al. found that among five different extracts, the ethyl acetate (EA) fraction exhibited the most potent antioxidant and anti-diabetic activities. 5-CQA derivatives and flavonoid glucosides were identified as the primary bioactive constituents. The anti-diabetic effect was primarily attributed to α-glucosidase inhibition, while the antioxidant activity was linked to free radical scavenging [140]. The extract of *Artemisia afra* Jacq. ex Willd. was shown to possess in vitro anti-diabetic activity via an α-glucosidase inhibition bioassay. The main bioactive compounds identified were 1,5-diCQA and 3,5-diCQA. Notably, the α-glucosidase inhibitory activity of these compounds surpassed that of acarbose, a standard anti-diabetic drug, providing strong support for the development of natural therapies for diabetes [141].

### 4.2. In Light Industry

Light industry encompasses the production of consumer goods, including food, textiles, leather, paper, and household chemicals. Within these sectors, the application of CQAs has been documented in several areas, most notably in animal husbandry, skin-whitening cosmetics, and food preservation.

In husbandry, the comprehensive ban on antibiotic use in China has spurred significant interest in natural plant extracts, such as CQAs, as potential alternatives. Consequently, research in this area has proliferated in recent years. In a study by Wei et al., dietary supplementation with 5-CQA in weaned piglets significantly enhanced serum concentrations of total protein, albumin, and total cholesterol compared to the control group. Furthermore, it increased the activity of alkaline phosphatase and superoxide dismutase in the ileal mucosa. Notably, 5-CQA also upregulated the expression of key genes involved in intestinal barrier function and antioxidant defense, including *ZO-1*, *SLC7A*, and *Nrf2*, in the duodenum [142]. Zhang et al. demonstrated that 5-CQA alleviates oxidative stress and intestinal inflammation in weaned piglets by suppressing the TLR4/NF-κB signaling pathway and concurrently activating the Nrf2 antioxidant pathway [143]. These findings indicate that 5-CQA enhances intestinal function and promotes growth performance, thereby providing a mechanistic foundation for the development of natural feed additives. The interplay between these two pathways is delineated in Figure 5. Furthermore, 5-CQA has been evaluated as a dietary supplement in broiler chickens. Research indicates that under high-density (HD) stocking stress, 5-CQA supplementation significantly ameliorates intestinal impairment by enhancing serum antioxidant capacity and improving jejunal villus development. These collective effects contribute to the restoration of intestinal barrier integrity [10]. Beyond its role in gut health, emerging research indicates that 5-CQA also protects against cadmium-induced neurotoxicity in chicken cerebral cortical neurons. This neuroprotective effect is mediated through the inhibition of the AMPK-ULK1 signaling pathway, as demonstrated in both in vivo and in vitro models [144]. In laying hens, dietary supplementation with 5-CQA at 250 mg/kg significantly improved key egg quality parameters, including eggshell thickness, egg weight, yolk color intensity, and Haugh units [145].

In the food preservation industry, CQAs have been reported to inhibit the proliferation of *Salmonella Enteritidis* in chilled chicken, thereby extending its shelf life by preventing microbial spoilage [146]. For cherry (*Prunus avium* L.), a representative of perishable foods, coating with a 5-CQA-based agar composite film, when activated by light irradiation, extended the postharvest storage period by 9 days [129]. This shelf-life extension is primarily attributed to the light-activated antimicrobial efficacy of the film, which generates ROS upon illumination to effectively inhibit microbial growth (Figure 5). Incorporating 5-CQA into a starch-whey protein-based packaging material significantly enhanced its antioxidant, UV-blocking, and antibacterial properties. In experiments with fresh bananas (*Musa acuminata* Colla), this 5-CQA-enriched packaging effectively suppressed browning [147]. When embedded into nanofibers for food packaging, 5-CQA demonstrated potent antioxidant activity and favorable release kinetics, highlighting its potential as a natural, non-toxic additive [148]. Furthermore, treatment with 5-CQA reduced the respiration rate and slowed the decline of soluble solids, firmness, and ascorbic acid content in ‘Lvbao melons’ during storage. A concentration of 30 mg/L was identified as the most effective, outperforming both 10 mg/L and 50 mg/L doses [149]. 5-CQA exhibits excellent inhibitory effects against gray mold (*Botrytis cinerea*). A zein-chitosan-chlorogenic acid (ZCC) nanoparticle delivery system was developed to leverage this property. The ZCC nanoparticles effectively controlled *B. cinerea* growth on grapes and kiwifruit. Separately, 5-CQA treatment delayed ripening and senescence in peach (*P. persica*) fruit, enhancing disease resistance and extending its storage life [150]. Song et al. developed an immobilized lipase (CSL@OMS-C18) for the enzymatic synthesis of acylated chlorogenic acid derivatives (ACDs). The immobilized enzyme exhibited excellent conversion efficiency, catalytic stability, and reusability. The study also evaluated the bioactivity of the resulting ACDs, providing a foundation for the sustainable enzymatic production of liposoluble antioxidant compounds for food applications [151]. Zhang et al. found that a composite antioxidant (300 ppm tocopherol + 200 ppm 5-CQA) effectively suppressed oxidation in algal oil emulsions. The mechanism involves 5-CQA facilitating the regeneration of tocopherol and modulating its distribution at the oil-water interface. This composite antioxidant did not compromise the physical stability of the emulsion, thereby supporting the bioaccessibility of the algal oil [152].

Within the skincare industry, several recent reports have highlighted the potential of CQAs. For instance, an anti-pollution film-forming spray (FFS), developed from coffee cherry flesh extract (FFS-CCS) whose key active constituents include 5-CQA, was clinically demonstrated to significantly improve skin hydration, reduce wrinkle appearance, and enhance skin tone, indicating its anti-aging efficacy [153] (Figure 5). Ruscinc et al. evaluated the protective effects of various polyphenols, including 5-CQA, against ultraviolet -induced lipid peroxidation in the stratum corneum. Their findings confirmed that 5-CQA possesses notable antioxidant and anti-inflammatory capacities. However, it is noteworthy that 5-CQA also exhibited pro-oxidant properties under specific conditions. This study underscores the multifaceted and context-dependent roles that polyphenols like 5-CQA play in skin protection [14].

### 4.3. In Plant Stress Resistance

Throughout their life cycle, plants are exposed to various biotic and abiotic stresses—including low temperature, salinity, drought, and pathogen infection—which can severely inhibit growth [83,154]. In response, plants have evolved sophisticated defense mechanisms to mitigate these adverse effects [154]. These adaptive strategies often involve the orchestrated accumulation of protective metabolites, regulated through complex signal transduction pathways [154,155,156]. Notably, recent studies have frequently reported that CQAs accumulate under stress conditions, enhancing the plant’s ability to resist such adversities [15,102,157].

Under cold stress, Xiao et al. investigated the molecular mechanisms of cold tolerance by comparing two citrus species with markedly different sensitivities [15]. Their metabolomic analysis revealed that 5-CQA and sphinganine specifically accumulated in the cold-hardy citrus species Ichang papeda (*C. ichangensis*) during cold stress, a phenomenon absent in the cold-sensitive HB pummelo (*Citrus grandis* (L.) Osbeck “Hirado Buntan”). Subsequent genes, *CiSPT* and *CiHCT2*, knockout and exogenous application experiments confirmed that these compounds are crucial for the cold resistance of Ichang papeda. The study concluded that 5-CQA functions as an antioxidant, directly scavenges H_2_O_2_ and O_2_^−^ to mitigate cold-induced cellular damage. They also noted that high accumulation of 5-CQA under cold stress has also been documented in the related species *Poncirus trifoliata* (L.) Raf. Based on this mechanistic understanding from [15], we have summarized the proposed cold tolerance pathway in Figure 5. In tomatoes (*S. lycopersicum*), which are susceptible to chilling injury during low-temperature storage, a study demonstrated that treatment with 50 mg/L 5-CQA effectively alleviated chilling injury and reduced weight loss. This treatment also improved cell membrane integrity and lessened oxidative damage, resulting in a significant 30–40% reduction in the incidence of chilling injury [157]. Interestingly, phenolic compounds such as CQAs exhibit a context-dependent dual role in plant physiology. In intact cells, CQAs are stored in the vacuole, while polyphenol oxidase (PPO) is localized in the cytoplasm, plastids, plasma membrane, and mitochondria [158,159]. Upon mechanical damage, phenol-enzyme regional distribution is disrupted, and the protective role of CQAs can reverse and mix with Polyphenol oxidase (PPO); CQAs act as direct substrates for PPO-catalyzed oxidation into highly reactive o-quinones [160,161]. These quinones subsequently undergo non-enzymatic polymerization into dark melanoidins, thereby accelerating browning [159,161]. For instance, contrary to its protective role in other species, CQAs, along with catechin, has been reported to accelerate the browning reaction in fresh-cut potatoes [162]. Conversely, another study on fresh-cut potatoes reported that exogenous application of CQAs delayed browning, reduced PPO activity, and inhibited quality deterioration in potato slices [163]. This seemingly paradoxical finding underscores the complex interactions among phenolic compounds, cellular integrity, and enzymatic activity, highlighting the profoundly context-dependent dual role of CQA. Understanding this duality is essential for comprehending the complex roles of plant phenolics. Furthermore, in Mango (*Mangifera indica* L.), Perveen et al. used phenolic acid profiling to show that salt stress triggered the significant accumulation of hydroxybenzoic acids, including a 510% increase in 5-CQA, in Mango cultivar ‘Bappakkai’ [164]. This accumulation was strongly associated with enhanced salt tolerance (Figure 5). In apple (*M. domestica*), Feng et al. reported that overexpression of *MdANR*, a key gene in the proanthocyanidin (PA) biosynthesis pathway, enhanced tolerance to heat and high-light stress in apple skin and callus tissues [165]. This genetic modification led to increased accumulation of both PAs and 5-CQA, which were found to reduce sunburn damage, a protective relationship modeled in Figure 5.

Plants exposed to metalloid arsenic (AS) often exhibit morphological, physiological, and growth-related abnormalities, collectively leading to reduced productivity [166]. In maize (*Z. mays*), both hesperidin (HP) and 5-CQA effectively mitigate the detrimental effects of AS stress. This includes alleviating the arsenic-induced loss of dry and fresh biomass, counteracting the negative impact of arsenic toxicity on PSII photochemical quantum efficiency (Fv/Fm), and restoring redox homeostasis within chloroplasts through HP- or CA-mediated increases in ascorbate and glutathione levels. These mechanisms collectively reduce the physiological and biochemical damage caused by AS in maize [166].

CQAs also play a significant role in plant defense against biotic stress. In rice, one study evaluated the resistance of two rice cultivars to WBPH (*S. furcifera*) by analyzing antixenosis, antibiosis, and tolerance mechanisms. Metabolomic analysis revealed that CQAs are key resistance compounds in the highly resistant cultivar KL35, significantly prolonging WBPH developmental time and reducing its fecundity. The study also demonstrated that exogenous application of CQAs to a susceptible cultivar, TN1, enhanced its resistance to WBPH, markedly reducing the insect’s survival rate and body weight. These findings highlight the considerable potential of plant-derived CQAs for agricultural applications [102] (Figure 4). Similarly, CQAs exhibit notable insecticidal activity in sweet potato. Liao et al. found that treating sweet potato leaves with exogenous CQAs significantly reduced SPW feeding. Further biochemical assays confirmed that compounds containing a 1-hydroxyquinoyl group inhibit SPW intestinal digestive enzymes and activity [75,103] (Figure 4). In eggplant (*Solanum melongena* L.), Kumar et al. observed significant variation in larval resistance to *Spodoptera litura* (Fabricius, 1775) among different cultivars. Untargeted metabolomics identified CQAs as the primary resistance compounds. Feeding larvae, a diet supplemented with 5-CQA resulted in a threefold reduction in larval mass, a twofold increase in mortality, and significantly decreased pupation and eclosion rates compared to the control group. Silencing the key CQAs biosynthetic gene *SmHQT* in the highly resistant cultivar RL22 reduced CQAs levels threefold and diminished its resistance to the armyworm, while exogenous CQAs application restored resistance. These results collectively demonstrate the larvicidal properties of CQAs against *S. litura* [167]. Furthermore, CQAs have been reported to confer resistance against another lepidopteran pest, *Mythimna separata* (Walker, 1865). Lin et al. observed a significant concentration-dependent lethal effect when third-instar larvae were fed 5-CQA. The study also assessed the sublethal effects of 5-CQA at the LC_20_ concentration, noting significantly reduced total survival, pupation rate, eclosion rate, sex ratio, and fecundity compared to the control. These results indicate that CQAs can effectively control *M. separata* populations [168].

### 4.4. Bioavailability, Metabolism, and Structure-Activity Relationships (SAR) of CQAs

Although the broad pharmacological profile of CQAs is well-documented, research on their bioavailability, metabolic fate, and structure–activity relationships (SAR) remains comparatively limited. Current evidence indicates that the absorption of CQAs in vivo occurs mainly via two pathways: (I) approximately one-third is absorbed intact in the stomach and upper gastrointestinal tract and enters systemic circulation directly; (II) the remaining portion largely reaches the colon intact, where it is extensively metabolized by gut microbiota, with the resulting metabolites subsequently absorbed [169,170,171,172,173,174]. Only trace amounts of the parent CQAs are ultimately excreted in urine [169,170].

Multiple clinical studies have delineated this intricate metabolic process. For example, Mariana et al. detected several CQAs isomers—including three CQAs and three diCQAs—in human plasma following coffee consumption, along with a range of urinary metabolites [171]. Similarly, Stalmach et al. identified numerous metabolites in plasma and urine, noting that despite considerable inter-individual variability, about one-third of orally administered CQAs are absorbed intact in the gastrointestinal tract [172,173]. The unabsorbed fraction almost completely reaches the colon, where gut microbiota transform it into various metabolites such as dihydrocaffeic acid (DHCA), dihydroferulic acid (DHFA), caffeic acid (CA), 5-CQA, gallic acid (GA), ferulic acid (FA), isoferulic acid (isoFA), vanillic acid (VA), sinapic acid, *p*-hydroxybenzoic acid, and *p*-coumaric acid (*p*-CoA) [171,172,174]. Additionally, some studies suggest that the colon may participate in the reabsorption of CQAs excreted into digestive juices [170]. Although metabolite profiles differ across studies—likely due to variations in analytical methods, dosage, and individual physiology—they consistently support the two primary bioavailability pathways of CQAs in vivo.

Once in systemic circulation, both parent CQAs and their microbial metabolites undergo further hepatic metabolism. The detection of substantial levels of methylated and sulfated conjugates in plasma and urine indicates extensive Phase II metabolism in the liver [173,175].

Regarding structure–activity relationships, several pharmacophores have been identified as critical: the catechol moiety in the caffeoyl group, the acrylic acid double bond, and the acylation pattern of the quinic acid moiety. Supporting this, Cao et al. reported that removing phenolic hydroxyls from the caffeic acid moiety abolished hypolipidemic activity, whereas modifications to the quinic acid portion had minimal impact [176]. Based on SAR analyses of analogs, Wang et al. designed and synthesized a photoaffinity-labeled probe molecule [125,176]. Moreover, dicaffeoylquinic acids often demonstrate enhanced potency, possibly due to a higher density of active caffeoyl groups [9].

In summary, current findings indicate that the in vivo activity of CQAs is closely linked to the caffeoyl group. The overall bioactivity likely results from the combined effects of a small fraction of intact CQAs, colonic microbial metabolites, and potential Phase II hepatic metabolites, with the caffeoyl moiety serving as the essential pharmacophore.

## 5. Summary and Perspectives

This review systematically consolidates the latest advances in the biosynthesis and multifaceted bioactivities of CQAs. As a class of naturally occurring phenolic acids, CQAs have attracted increasing research interest due to their broad pharmacological properties and industrial applicability. However, the persistent ambiguity in nomenclature—particularly the historical conflation of 3-CQA and 5-CQA as “chlorogenic acid”—remains a barrier to the integration and communication of scientific findings. We therefore emphasize the urgent need for standardized nomenclature and encourage the consistent use of IUPAC-recommended names and CAS numbers in future studies.

The bioactivity profile of CQAs underpins their potential across diverse sectors. Pharmacologically, CQAs exhibit anti-inflammatory, antioxidant, neuroprotective, anticancer, antimicrobial, antiviral, and antidiabetic properties, often mediated through key pathways such as NF-κB, TLR, and Nrf2. In light industry, CQAs serve as antibiotic alternatives in animal feed, natural preservatives in food packaging, and active ingredients in skincare formulations due to their antioxidant and UV-protective properties. Moreover, CQAs contribute to plant resilience against cold, drought, salinity, and insect herbivory, primarily through ROS scavenging and direct antimicrobial or antifeedant actions.

Despite these advances, critical research gaps persist, charting a clear course for future investigations. A primary unresolved area is the biosynthesis of poly-acylated CQAs (e.g., diCQAs, triCQAs), where the specific enzymatic pathways, regulatory logic, and the role of non-enzymatic acyl migration in vivo remain poorly characterized and warrant elucidation. Another significant bottleneck lies in our limited understanding of CQA transport and compartmentalization, particularly the identification of vacuolar transporters responsible for their storage. To construct a comprehensive regulatory network for CQA biosynthesis, future efforts should leverage integrated systems biology approaches, combining genomics, transcriptomics, proteomics, and metabolomics, especially in non-model medicinal plants. Furthermore, despite promising preclinical results, systematic toxicological evaluations, in vivo efficacy studies, and clinical translation of CQAs are still scarce.

Looking ahead, the biotechnological exploitation of CQA pathways holds substantial promise. Metabolic engineering of engineered microbes (e.g., *E. coli*, *S. cerevisiae*) or plant systems to express key biosynthetic genes offers a sustainable platform for the large-scale production of specific CQAs, reducing reliance on plant extraction. Synthetic biology strategies, involving the construction of synthetic gene clusters and optimization of chassis cells via modular pathway engineering, could enable the tailored production of rare or structurally complex CQAs with enhanced bioactivities. In agriculture, genome editing techniques like CRISPR/Cas9 applied to transcriptional regulators or structural genes could pave the way for developing stress-resistant crops with elevated CQA levels, combining improved agronomic traits with added health benefits. Finally, incorporating biotechnologically produced CQAs into functional foods, nutraceuticals, and phytopharmaceuticals represents a growing market opportunity, ensuring purity, scalability, and sustainability.

In conclusion, while significant progress has been made in understanding the biosynthesis and bioactivity of CQAs, leveraging modern biotechnological tools and interdisciplinary approaches to address the existing knowledge gaps will unlock their full potential in medicine, agriculture, and light industry. We advocate for interdisciplinary collaborations to translate these promising natural compounds from bench to market.

## Figures and Tables

**Table 1:** CQAs Derivative References.

Number	Systematic Name (with Common Aliases)	CAS Number
Mono-caffeoylquinic acids		
1	1-O-Caffeoylquinic acid (1-CQA)	1241-87-8
2	5-O-Caffeoylquinic acid (Chlorogenic acid, 5-CQA)	327-97-9
3	4-O-Caffeoylquinic acid (Cryptochlorogenic acid, 4-CQA)	905-99-7
4	3-O-Caffeoylquinic acid (Neochlorogenic acid, 3-CQA)	906-33-2
Di-caffeoylquinic acids		
5	1,3-Dicaffeoylquinic acid (1,3-diCQA)	30964-13-7
6	1,4-Dicaffeoylquinic acid (1,4-diCQA)	1182-34-9
7	1,5-Dicaffeoylquinic acid (1,5-diCQA)	212891-05-9
8	3,4-Dicaffeoylquinic acid (isochlorogenic acid B (3,4-diCQA)	14534-61-3
9	3,5-Dicaffeoylquinic acid (isochlorogenic acid A (1,3-diCQA)	2450-53-5
10	4,5-Dicaffeoylquinic acid (isochlorogenic acid C (4,5-diCQA)	57378-72-0
Tri-caffeoylquinic acids		
11	1,3,4-Tricaffeoylquinic acid (1,3,4-triCQA)	1073897-77-4
12	1,3,5-Tricaffeoylquinic acid (1,3,5-triCQA)	150035-89-5
13	1,4,5-Tricaffeoylquinic acid (1,4,5-triCQA)	1073897-83-2
14	3,4,5-Tricaffeoylquinic acid (3,4,5-triCQA)	86632-03-3
Tetra- caffeoylquinic acids		
15	1,3,4,5tetracaffeoylquinic acid (1,3,4,5-tetraCQA)	158364-86-4

## Data Availability

No new data were created or analyzed in this study. Data sharing is not applicable to this article.
